# Minding the Prevention Protocol for Blood-Borne Diseases via EM Residents

**DOI:** 10.5812/traumamon.9380

**Published:** 2013-05-26

**Authors:** Soad Mahfoozpour, Alireza Baratloo, Hamidreza Hatamabadi, Kiandokht Karimian, Saeed Safari

**Affiliations:** 1 Safety Promotion and Injury Prevention Research Center, Shahid Beheshti University of Medical Sciences, Tehran, IR Iran; 2 Department of Emergency Medicine, Shohadaye Tajrish Hospital, Shahid Beheshti University of Medical Sciences, Tehran, IR Iran; 3 Imam Hosein Hospital, Shahid Beheshti University of Medical Sciences, Tehran, IR Iran; 4 Department of Emergency Medicine, Vali Asr Hospital, Arak University of Medical Sciences, Arak, IR Iran

**Keywords:** Preventive Health Services, Epidemiology, Prevention and Control

## Abstract

**Background:**

The emergency department (ED) is one of the high-risk places for blood-borne disease (BBD) transmission.

**Objectives:**

This study aimed to assess the rate of adherence to preventive measures (PM) against the blood-borne diseases via emergency medicine (EM) residents.

**Materials and Methods:**

In this descriptive cross-sectional study, 80 emergency residents of an educational public hospital were observed with regard to abiding by the preventive measures from March to May 2010.

**Results:**

Cleaning blood tainted skin before phlebotomy or IV line preparation and hand washing before donning latex gloves achieved the maximum (88/150) and minimum (0/150) scores. The most observed barriers of PM were frequent attendance of patients (85%), work load (80%), and need to work rapidly (68%).

**Conclusions:**

It seems that, the preventive instructions are not optimally respected by ERs possibly due to the crowded wards, high work load and the need to work rapidly.

## 1. Background

The increasing frequency of blood-borne diseases (BBD) among physicians, indicates the need for developing preventive strategies to reduce the burden of these diseases (1, 2). The emergency department (ED) is one of the high-risk places for BBD transmission of the preventable BBD (3). The high burden of BBD despite vaccination for some infections such as HBV is of concern (1). Almost always, it is not possible to determine the high-risk patients in the ED thus, preventive measures such as using gloves, eye shield, mask, gowns and prohibition of reused instruments can be helpful (4).

## 2. Objectives

This study aimed to assess the rate of adherence to preventive measures (PM) against the blood-borne diseases via emergency medicine residents.

## 3. Materials and Methods

In this descriptive cross-sectional study, 80 emergency medicine residents (EMR) of Imam Hossein Hospital (in Tehran, Iran) were observed with regard to attention to preventive measures against BBD from March to May 2010. All emergency medicine residents at Imam Hossein Hospital were studied. The study protocol was accepted by the ethics committee of, and the principles of the Declaration of Helsinki were applied throughout the study. A designed observation form was structured in two sections according to global preventive instructions for data collection. The observation form was designed by cooperation of two specialists of infectious diseases and one epidemiologist. Reliability was assessed using the Cronbach alpha coefficient. Cronbach's alpha coefficient equals 0.76. The first section included 13 items such as hand washing before and after procedures, wearing gloves, glasses, and gowns while preparing IV lines, and considering safety issues while using needles and sharp objects. Each Item, based on the quality of performance was divided to poor (not done), fair (incompletely done) and good (completely done). Then for scoring each item, 0 score for poor performance, 1 for fair and 2 for good were considered (Table 1). The second section included 10 items related to preventive instructions of BBD. After the assessment was completed by the observer, the second part of the questionnaire was prepared for the residents to respond to (Table 2). Personal and occupational characteristics were also recorded. Content validity of instruments for the data collection was confirmed by inter-rater reliability (κ= 0.85). For each sample, four observations were performed by one observer, from which, the first one was not considered in the research results, but the next three observations were recorded. Finally data analysis was performed by SPSS version 18. Statistical analysis using Mann-Whitney and Spearman tests was done. P <0.05 was considered significant.

**Table 1.** Minding Preventive Protocol for Protection Against Blood-Borne Diseases

Preventive Instructions Performance	Mean Score^a^	SD	Poor, No. (%)	Fair, No. (%)	Good, No. (%)
**Hand Washing Before DonningLatex Gloves**	0	0	80 (100)	0 (0)	0(0)
**Use of Latex Glove Before Medical Procedures**	95	20	4 (5.0)	0 (0)	76(95.0)
**Hand Washing After Degloving**	38	37	45 (56.3)	9 (11.2)	26(32.5)
**Wearing Gown When Secretion and Blood May Be Splattered**	6	23	57 (93.4)	1 (1.6)	3(4.9)
**Wearing Mask When Secretion and Blood May Be Splattered**	10	30	71 (89.9)	0 (0)	8(10.1)
**Wearing Glass When Secretion and Blood May Be Splattered**	3	16	75 (997.4)	0 (0)	2(2.6)
**Distribution of Secretion and Blood After IV Line Preparation, Angiocatheter Removal, Phlebotomy or Suction**	77	43	10 (23.3)	0 (0)	33(76.7)
**Cleaning the Bloody Skin Before Phlebotomy or IV Line Preparation With Povidone-Iodine or Alcohol**	88	32	5 (11.6)	0 (0)	38(88.4)
**Needle Bending After Phlebotomy**	5	22	37 (94.9)	0 (0)	2(5.1)
**Recapping the Needle After Phlebotomy or IV Injection**	3	16	38 (97.4)	0 (0)	1(2.6)
**Inserting the Needle andAngiocatheterin Safety Box**	3	16	39 (97.5)	0 (0)	1(2.5)
**Use of Safety Box**	3	16	39 (97.5)	0 (0)	1(2.5)
**Use of Bandage in Presence of Wound**	6	24	75 (93.8)	0 (0)	5(6.2)

^a^0 score considered for poor performance, 1 for fair and 2 for good; Maximum and minimum score was 150 and 0 respectively

**Table 2.** Excuses forNot Minding Preventive Instructions Against Blood-Borne Diseases

Excuses	Not Selected^b^, No. (%)	Selected^b^, No. (%)	Selection Order ^a^, No. (%)			
			I	II	III	IV
**Work Shift**	36 (90)	4 (10)	2 (5)	1 (3)	1 (3)	0 (0)
**High Number of the Admitted Patients**	6 (15)	34 (85)	21 (53)	11 (28)	1 (3)	1 (3)
**Need for Rapid Performance**	13 (33)	27 (68)	1 (3)	22 (55)	4 (10)	0 (0)
**High Work Load**	8 (20)	32 (80)	4 (10)	10 (25)	16 (40)	2 (5)
**High Work Hours**	29 (73)	11 (28)	1 (3)	4 (10)	3 (8)	3 (8)
**Inappropriate Ward Construction**	36 (90)	4 (10)	1 (3)	0 (0)	2 (5)	1 (3)
**Job Experience**	38 (95)	2 (5)	0 (0)	1 (3)	1 (3)	0 (0)
**Job Satisfaction**	40 (100)	0 (0)	-	-	-	-

^a^Order of selection in questionnaire based on degree of importance

^b^Selected by objects as a barrier

## 4. Results

Among 15 evaluated preventive instruction measures in this study, three items with the highest scores were, 1) wearing latex gloves before medical procedures, 2) cleaning blood-stained skin before phlebotomy or IV line preparation with povidone-iodine or alcohol, and 3) no peripheral splatter of blood and secretions after IV line preparation, angiocatheter removal, or suctioning (Table 1). Hand washing before gloving was not done by anyone. Gowning, use of a mask and eyewear was done by 3.8%, 10%, and 25% of physicians respectively. Caring for prevention of blood and secretions splatter after IV line preparation, angiocatheter removal, phlebotomy or suctioning was done by 41.3% of physicians. There was no statistically significant difference between the scores of those respecting preventive measures and gender (P > 0.05). Hand washing after degloving was the only measure respected more by the senior residents compared with juniors ; its score was 16.7 for first year, 27.8 for second year, and 46.8 for third year residents (P = 0.003). The total score for respecting instructions for BBD also increased according to residency year and was 19.3%, 23.3% and 29.5% for the first, second and third year of residency respectively (P = 0.002). Hand washing after glove removal differed between IV line preparation and intubation (47 vs. 25 score) (P = 0.013). The mean total score of respecting instructions to prevent BBD did not differ according to the procedure (P = 0.065). There was no statistically significant difference with regard to respecting the preventive protocol items between day and night shift physicians (P > 0.05). The most common causes for disrespecting preventive instructions for BBD were the large number of patients and crowded wards, high work load, and the need to work rapidly (Table 2).

## 5. Discussion

Determining the cause for disrespecting BBD preventive measures is an issue of importance. This study showed that the most common barriers for respecting preventive instructions for BBD were the large number of patients and crowded wards, high work load, and need to perform duties rapidly. The age, sex, and residency year did not contribute to non-adherence to preventive instructions. A study by Bagheri et al. (5) assessed 65 nurses working at emergency wards showed that the majority of these nurses moderately respected the preventive strategies, but our study showed a better performance among emergency medicine residents. The nurses were reported to have difficulties wearing gloves. Forgetting to wear mask or eyewear, hand washing before nursing procedures and removing the needles from the safety boxes and lack of positive attitude toward the necessity to respect these preventive protocols were the main barriers for not minding the preventive protocols for blood-borne diseases. Many of these barriers were also reported by emergency medicine residents. High work load, nonexistence of obligations to abide by preventive strategies in the wards, need for rapid performance and long working hours, lack or insufficiency of facilities, and high work load in the wards were the other reported excuses by nurses, which also were mentioned by the emergency medicine residents in the current study. Moro et al. (6) showed that 98 out of 440 healthcare workers (22.3%) reported to have needle stick exposure during the previous year. Unsafe recapping was observed in 98 percent of injections in hospitals, and 12 percent in clinics; the related frequency in this study was 25 percent, showing the necessity for training courses for emergency medicine residents. Van Gement-Pijnen et al. (7) evaluated the performance of preventive strategies by nurses, physicians, and laboratory personnel. The results showed that research subjects were knowledgeable but they had poor performance; therefore, they required more training.

Our study also showed the necessity to conduct training courses. Leiss et al. (8) reported the necessity of training courses and improvement of technical abilities in health care personnel, which is congruent with the present study. Also, Simbar et al. (9) evaluated 58 obstetricians and found that 96.6 percent of them had good knowledge and only 29.3 percent had good performance in minding preventive measures for AIDS. They reported a reverse association between performance of subjects and the years of their clinical experience. On the contrary, our study showed that as residency seniority increased, the performance improved.

It may be concluded that the preventive protocol for BBD were not abided by emergency medicine residents as expected; this might be due to the large number of patients and crowded wards, high work load, and need for fast performance of duties. It is prudent to develop training courses to improve the knowledge and performance of emergency medicine residents.
